# The prevalence of antimicrobial resistance and carriage of virulence genes in *Staphylococcus aureus *isolated from food handlers in Kuwait City restaurants

**DOI:** 10.1186/1756-0500-2-108

**Published:** 2009-06-16

**Authors:** Edet E Udo, Siham Al-Mufti, M John Albert

**Affiliations:** 1 Department of Microbiology, Faculty of Medicine, Kuwait University, Kuwait; 2 Public Health Laboratory, Ministry of Health, Kuwait

## Abstract

**Background:**

*Staphylococcus aureus *is a major cause of food poisoning due to their ability to produce enterotoxins which if ingested in sufficient amounts results in sickness. Food handlers carrying enterotoxin-producing *S. aureus *in their noses or hands can contaminate food leading to food poisoning. We characterized 200 *S. aureus *obtained from food handlers in different restaurants for antibacterial resistance and the carriage of virulence genes.

**Findings:**

Susceptibility to antibacterial agents was determined by disk diffusion and Etest. PCR was used to detect genes for accessory gene regulator (agr); capsular polysaccharide (cap) 5 and 8, staphylococcal enterotoxins (SE), toxic shock syndrome toxin-1 (TSST-1) and Panton-Valentine leukocidin (PVL). Isolates were typed using pulsed-field gel electrophoresis. In total 185 (92.5%) of the 200 isolates expressed resistance to antibacterial agents. They were resistant to penicillin G (82.0%), tetracycline (19.0%), erythromycin (2.5%), clindamycin (2.0%), trimethoprim (7.5%), kanamycin (2.5%), streptomycin (1.5%), ciprofloxacin (1.5%), fusidic acid (1.0%) and cadmium acetate (68.0%). Seventy-six (38.0%) and 114 (57.0%) isolates had type 5 and type 8 capsular polysaccharides respectively. The agr types I, II and III alleles were detected in 50.5%, 20.0% and 23.5% of the isolates respectively. They contained genes for SEI (38.5%), SEG (24.0%), SEC (23.0%), SEB (12.5%), SEH (21.5%), SEA (11.0), SED (1.5%), SEE (1.5%), TSST-1 (4.0%) and PVL (9.0%).

**Conclusion:**

This study revealed a high prevalence of antibacterial resistance and virulence determinants in *S. aureus *from food handlers in Kuwait restaurants justifying the screening of food handlers to detect and treat carriers and protect restaurant customers from staphylococcal food poisoning.

## Background

*Staphylococcus aureus *can cause localized and invasive infections in humans. This is attributed to its ability to produce a variety of virulence factors such as capsular polysaccharides, staphylococcal enterotoxins (SEs), toxic shock syndrome toxin 1 (TSST-1) [1,2], panton – valentine leukocidin (PVL) [3] and accessory gene regulators (agr) [1,4-6]. Although *S. aureus *isolates produce one of 11 serotypes of capsular polysaccharides, most clinical isolates belong to serotypes 5 and 8 [2]. Enterotoxin-producing *S. aureus *are common causes of food poisoning in many parts of the world. The ingestion of the preformed toxins in food often leads to the development of food poisoning. The symptoms typically have a rapid onset (2–6 h) and may include nausea, vomiting, diarrhea and abdominal pain [1]. Nasal and hand carriage of enterotoxigenic *S. aureus *by food handlers is an important source of staphylococcal food contamination in restaurants and fast food outlets [7]. Therefore it is important to detect *S. aureus *carriage among food handlers to prevent possible food contamination by them resulting in food poisoning.

Food poisoning outbreaks result in huge financial losses to restaurants, in addition to the loss of reputation and confidence among the public. Staphylococcal food-borne diseases are estimated to cause 6 – 81 million illnesses and up to 9000 deaths, and accounts for 14–20% of outbreaks involving contaminated food in the USA [8].

Most of the studies on *S. aureus *associated with food poisoning have focused on screening of the isolates for enterotoxins [1,9-13] with only sparse data on the carriage of other virulence factors and antimicrobial resistance among *S. aureus *obtained from food handlers [14-17] especially in the Arabian Gulf countries. Although we previously studied the prevalence of staphylococcal enterotoxins in *S. aureus *isolated from food handlers in Kuwait city [10], their susceptibility to antibacterial agents were not investigated. Therefore in the present study, we characterized *S. aureus *isolated from food handlers in Kuwait City restaurants for their susceptibility to antibacterial agents and the carriage of virulence associated genes.

## Methods

### *S. aureus *isolates

In total, 200 *S. aureus *isolates were recovered from food handlers working in 50 Kuwait City restaurants from 2003 to 2005. They consisted of 133 (102 isolates from nasal and 31 hand swabs) of 500 swabs from 250 adult male workers in 50 restaurants, during screening of food handlers as demanded by the City Council, yielding a carriage rate of 53.2%. The Kuwait Municipal Council mandated a compulsory screening of restaurant workers to detect *S. aureus *carriers. Only one sample was investigated from the same food handler. The study also included 67 isolates obtained from 31 stool samples, 9 throat swabs and 27 nasal samples obtained from food handlers during routine investigations of suspected cases of food poisoning in different restaurants. Bacteria were isolated and identified using standard bacteriological methods including cultural characteristics, Gram stain, catalase, tube coagulase and DNase tests [10]. The isolates were preserved in skimmed milk at -80°C.

### Susceptibility to antibacterial agents

Susceptibility to antimicrobial agents was determined by the disk diffusion method [18] Disks containing cadmium acetate (50 μg), propamidine isethionate (100 μg), and mercuric chloride (109 μg) were prepared in the laboratory [19]. A zone diameter of 6–10 mm indicated resistance to cadmium acetate, propamidine isethionate and mercuric chloride. The minimum inhibitory concentrations (MICs) of methicillin, vancomycin and teicoplanin were determined with Etest strips (AB Biodisk, Solna, Sweden) according to the manufacturer's instructions. *S. aureus *strain ATCC25923 was used for quality control.

### Isolation of DNA for PCR amplification

DNA for PCR was extracted as described previously [3]. The extracted DNA was used for PCR immediately or stored at 4°C and used within three months.

### Detection of genes for staphylococcal enterotoxin (SE) and toxic shock syndrome toxin (TSST-1)

PCR amplification was performed for genes encoding staphylococcal enterotoxins SEA (*sea*), SEB (*seb*), SEC (*sec*), SED (*sed*), SEE (*see*), SEG(*seg*), SEH (*seh*), and SEI (*sei*) and toxic shock syndrome gene TSST-1(*tst*) with primers published previously [13,20] in three multiplex (MT) assays as follows: MT1 (*sec, sei *and *sed*), MT 2 (*seg, seh *and see) and MT 3 (*sea, seb *and *tst*). Based on optimization experiments, the primer concentrations used were 0.1 μM for *sea, sec*, and *tst*, and 0.2 μM for *seb, see, sed, seg *and *seh*. Amplifications comprised an activation step at 95°C for 15 min followed by 37 cycles of 94°C for 2 min, 51°C for 1 min and 72°C for 1 min with a final extension at 72°C for 7 min. Control strains used were *S. aureus*; ATCC13565 (SEA), ATCC14458 (SEB), ATCC23235 (SED), ATCC27664 (SEE), ATCC51811 (SEH), WBG525 (SEC, TSST-1), C90 (SEI) [16], C29 (SEG). The 16S rRNA primers, 5'-GGA GGA AGG TGG GGA TGA CG-3' and 5'-ATG GTG TGA CGG GCG GTG TG-3' [21] were used as internal controls. All tests were considered positive if the positive controls and the internal controls yielded the expected results. Negative controls for all PCR consisted of the PCR mixes without template DNA. PCR products (5 μl) were loaded in 2.0% (w/v) molecular biology grade agarose (BioRad, Hercules, USA) gels. PCR products were analyzed by agarose gel electrophoresis.

### Detection of genes for accessory gene regulators (agr), capsular polysaccharides (cap) and Panton-Valentine Leucocidin (lukS-lukF)

All isolates were tested for the presence of genes for PVL, agr types I, II, III and IV and cap type 5 and type 8 in PCR assays as described previously [3,22,23]. The 16S rRNA primer [21] was used as internal control in PCR amplifications.

### Pulsed-field gel electrophoresis (PFGE)

Counter-clamped homogeneous electric field (CHEF) electrophoresis of *Sma *I-digested chromosomal DNA was performed as described previously [19].

### Statistics

Differences in the distribution of virulence determinants between groups were assessed by Chi square test. A P-value of = 0.05 was considered significant.

## Results

One hundred and eighty five (92.5%) of the 200 *S. aureus *isolates were resistant to antibacterial agents. The majority, 164 (82.0%) and 136 (68.0%) of them were resistant to penicillin G and cadmium acetate respectively. Forty-two isolates (21.0%) were resistant to tetracycline, 15 isolates (7.5%) were resistant to trimethoprim and six isolates (3%) were resistant to kanamycin. Five and four isolates were resistant to erythromycin and streptomycin respectively. Two isolates were resistant to fusidic acid and only one isolate was resistant to methicillin and gentamicin. They were all susceptible to mupirocin, rifampicin, vancomycin, teicoplanin and linezolid. Sixty-five percent of them were resistant to two or more antibacterial agents and 23% were multiresistant with resistance to three or more classes of antibacterial agents.

The distribution of genes for capsular types and agr allotypes is summarized in Table 1. Whereas 114 isolates had gene for cap8, 76 isolates had gene for cap5. Ten isolates yielded negative results for both capsular polysaccharides. Only one agr type was detected in each isolate. One hundred and one isolates were agr type I, 47 isolates were type III, and 40 isolates were type II. None was positive for agr type IV. Twelve isolates yielded negative results for the four agr types tested.

**Table 1:** Distribution of genes for virulence factors in 200 *S. aureus *isolated from food handlers.

	Number (%) of isolates positive for virulence genes							
	
Sources of *S. aureus *(#)	*SE*	*cap5*	*cap8*	*agr I*	*agr II*	*agrIII*	*lukS-lukF*	*tst*
Nose (129)	93 (72.1)	49 (38.0)	72 (55.8)	67 (51.9)	24 (18.6)	28 (21.7)	11 (8.5)	7 (5.4)

Stool (31)	21 (67.7)	15 (48.4)	15 (48.4)	17 (54.8)	6 (19.3)	7 (22.5)	4 (12.9)	1 (3.2)

Hands (31)	22 (70.9)	9 (29.0)	21 (67.7)	13 (41.9)	6 (19.3)	11 (34.0)	3 (9.7)	ND

Throats (9)	6 (66.6)	3 (33.3)	6 (66.6)	4 (44.4)	4 (44.4)	1 (11.1)	ND	ND

Total (200)	142 (71.0)	76 (38.0)	**114 (57.0)**	101 (50.5)	40 (20.0)	47 (23.5)	18 (9.0)	8 (4.0)

**Abbreviations**: *cap*, genes for capsular polysaccharide, *agr*, genes for accessory gene regulator, *luKS-lukF *genes for PVL, *tst*, genes for TSST-1. ND, Not detected

**Figure 1:**
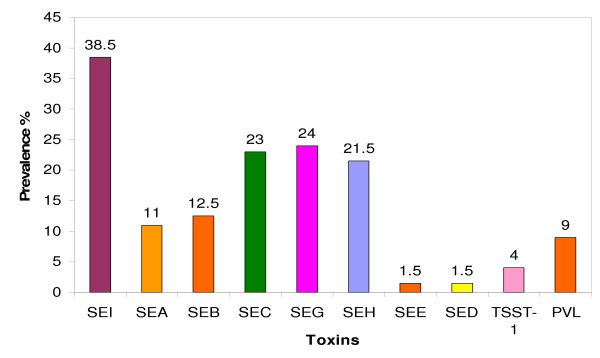
**Distribution of toxins in 200 *S. aureus *isolated from food handlers**.

The distribution of genes for SE, TSST-1 and PVL is summarized in Figure 1 and Table 2. In total, 142 (71.0%) isolates were positive for SE, 8 (4.0%) were positive for TSST-1 and, 18 isolates, including the only MRSA isolate, were positive for PVL. Gene for SEI was the most common SE gene. It was detected in 77 isolates. Fifty isolates contained single SE genes, 54 isolates carried two SE genes, 28 and 10 isolates contained three and four SE genes respectively.

**Table 2:** Enterotoxin gene profiles of the 142 enterotoxin-positive *S. aureus *from food handlers.

	Enterotoxin profiles
1	***S aureus *isolates with single SE genes (50)** *sea *(6), *seb *(9), *sec *(13), *seg *(9), *seh *(6), *sei *(7)

2	***S. aureus *isolates with two SE genes (54)** *seg, sei *(13); *seh, sei *(17); *sea, sec *(4); *sec, seg *(4); *sec, seh *(4); *seb, seg *(3); *sea, sei *(2); *seb, sei *(2); *seg, sed *(1); *seb, sec *(1); *sec, sei *(1); *see, sei *(1); *seb, see *(1).

3.	***S. aureus isolates with three SE genes (28)*** *sec, seg, sei *(9); *sec, seh, sei *(5); *sea, seg, sei *(4); *seb, seg, sei *(3); *sea, seh, sei *(3); *seb, seh, sei *(2);; *sec, sed; seg *(1); *seb, sec, seg *(1).

4	***S. aureus isolates with four SE genes (10)*** *sea, sec, seg, sei *(3); *sea, sec, seh, sei *(1); *seb, seg, seh, sei *(1); *seb, seg, seh, sei *(1); *seb, see, seh, sei *(1); *sea, seg, seh, sei *(1); *sea, sec, sed, seh*(1); *seb, sec, seh, sei *(1).

**Figure 2:**
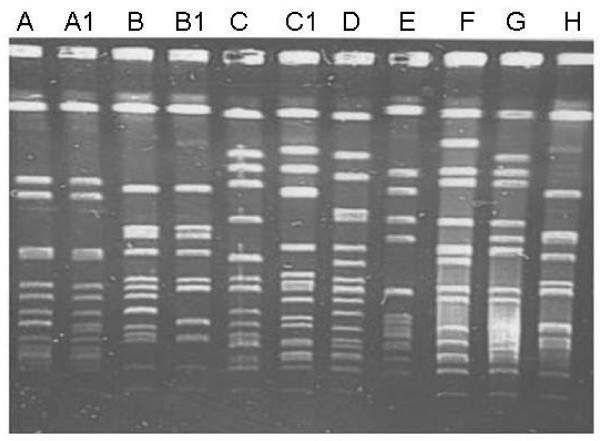
**PFGE of representatives of 18 PVL positive *S. aureus *from food handlers**. Lanes A: PFGE type A, Lane A1: PFGE type A1, Lane B: PFGE type B, Lane B1: PFGE type B1, Lane C: PFGE type C, Lane C1: PFGE type C1, Lane D: PFGE type D, Lane E: PFGE type E, Lane G: PFGE type G, Lane H: PFGE type H.

The characteristics of the 18 *lukS-lukF *positive isolates are presented in Table 3. They had different resistance patterns, belonged to different agr and cap types and harbored different SE genes but none carried gene for TSST-1. When typed by PFGE to determine if they were related, they yielded eight PFGE patterns designated types A to H (Figure 2, Table 3). Eight isolates obtained from stool, hand and nasal samples had the same PFGE pattern (type A) and 10 isolates belonged to seven PFGE patterns.

**Table 3:** Characteristics of 18 *S. aureus *isolates containing *lukS-lukF*

Isolates	Source	Resistance pattern	*agr *class	*cap *type	SE genes	PFGE pattern
33N	Nose	Pc, Tc, Tp, Cd	III	8	*sea, sei*	A
78N	Nose	Mc, Pc, Km, Tc, Fd, Cd	III	8	ND	A

157N	Nose	Pc, Km, Sm	III	8	*Seg*	A

342N	Nose	Cd	III	8	*Sei*	A1

515N	Nose	Cd	III	8	ND	H

42N	Nose	Pc,	I	5	*seb, seh, sei*	B

46N	Nose	Pc, Sm	I	5	*seh, sei*	C

268N	Nose	Cd	I	8	ND	C

298N	Nose	Pc	I	5	*seh*	C

489N	Nose	Pc, Cd	I	5	*seh, sei*.	E

666N	Nose	Pc, Tc, Tp, Cd	I	5	*sec, seg, sei*.	G

9S	Stool	Pc, Tp, Fd	III	8	*sea, seh, sei*	A

10S	Stool	Pc, Tp	III	8	*sea, seg, sei*	A

58S	Stool	Pc, Tp, Cd	III	8	*sea, sec, seg, sei*	A

76S	Stool	None	III	8	*Seb*	D

4S	Hand	Cd	III	8	ND	F

515S	Hand	Pc, Tc, Cd	III	8	*seh, sei*	A

666S	Hand	Pc, Tc, Tp, Cd	I	5	*sec, seg, sei.*	B

**Abbreviations**: Cd, cadmium acetate, Fd. fusidic acid. Km, kanamycin, Mc, methicillin, Pc, benzyl penicillin, Sm, streptomycin, Tc, tetracycline, Tp, trimethoprim, ND, not detected, *agr*, accessory gene regulator class, *cap*, capsular polysaccharide serotype type.

When the isolates obtained during the screening exercise (screening samples) were compared with those obtained during routine investigation of food poisoning cases (routine samples), the results were not significantly different for the distribution of genes for agr and capsular polysaccharides (data not shown). Similarly, 72.9% of the screening sample isolates (97/133) and 67.1% (45/67) of the routine samples isolates contained genes for SEs showing no significant difference in the distribution of genes for SE between the two sets of isolates (P value = 0.50). Furthermore *tst *was detected in 5.2% (7/133) of the screening samples isolates and in 1.5% (1/67) of the routine sample isolates (P value = 0.4)

## Discussion

This study has demonstrated a high prevalence of antibacterial resistance and diversity in the carriage of virulence genes in *S. aureus *obtained from food handlers employed in restaurants in Kuwait City. Our results that 92.5% of the isolates expressed resistance to antibacterial agents is similar to the prevalence of antibacterial resistance in methicillin-susceptible *S. aureus *obtained from patients in Kuwait hospitals [24] and to that found in *S. aureus *isolates from food handlers in Chile [11] and Botswana [17]. Although only one isolate was methicillin-resistant, the finding is significant because food handlers carrying MRSA had initiated outbreaks in hospitals in the Netherlands [14], USA [25] and Brazil [15]. As food handlers represent a section of the healthy population in the community, besides working in restaurants, the detection of high prevalence of antibiotic resistance in *S. aureus *isolated from them also highlights the growing problem of antibiotic resistance in the community.

The ability of *S. aureus *to colonize or infect its host is related to its ability to express virulence factors that facilitate their adherence to surfaces, cause damage or its ability to evade host's immune system [1,2]. Our isolates carried genes for a range of virulence factors including three of the four accessory gene regulators that regulate staphylococcal virulence factors [6]. Our result that 50.5% of the isolates were agr type I concurs with results of other studies that the majority of *S. aureus *from clinical sources belong to agr type I [4,23]. Similarly, our results that 57% of the isolates belonged to serotype 8 and 38% belonged to serotype 5 are in agreement with previous studies that showed that the majority of *S. aureus *obtained from clinical samples belonged to capsular polysaccharide serotypes 5 or 8 [2,23].

With regards to the carriage of SE determinants, the results of this study differ from that of a previous study in Kuwait [10]. The 71.0% prevalence of SE genes in this study was lower than the 86.6% prevalence detected previously in *S. aureus *from food handlers in Kuwait restaurants [10]. However, it was similar to the prevalence of 74.1% obtained from *S. aureus *isolated from food poisoning cases in Taiwan [26] and higher than the prevalence of SE reported in *S. aureus *from food handlers in Chile [19%, [11]] and from humans, food and animal sources in Malaysia [20.8%, [27]]. In addition, the demonstration of SEI as the most common SE gene in this study contrasted with the results of a previous study of SE in *S. aureus *isolated from food handlers in Kuwait restaurants [10] and other studies [9,11,12] where SEA was the most common SE. Furthermore, whereas 64.7% of the SE positive isolates in this study carried genes for two to four SEs, only 8.6% of *S. aureus *in the previous study from Kuwait carried more than one SE gene [10]. This is consistent with recent reports in the literature that also document the carriage of multiple SE gene in enterotoxigenic *S. aureus *[9,12,13]. The detection of multiple SE genes in recent *S. aureus *isolates could be due to the improvement in detection methods following the application of PCR technology and the discovery of new SE genes which were not tested in the previous study. It could also be due to frequent horizontal transmission of phage-mediated toxin genes among the staphylococcal populations [21].

PVL is widely distributed among some clones of community-associated MRSA (CA-MRSA) isolated in Kuwait and other countries [3,28,29] and was thought to contribute to the increased virulence of CA-MRSA isolates [3,28]. However, PVL has recently also been detected in healthcare-associated MRSA and in methicillin-susceptible *S. aureus *from patients [29]. Our report is the first in *S. aureus *obtained from food handlers in Kuwait and elsewhere. Its detection in 9.0% of the isolates in this study supports the suggestion that PVL-bearing phages are widespread among *S. aureus *of different genetic backgrounds and are not a specific characteristic of CA-MRSA [19,29]. The observation that eight of the 18 *lukS-lukF *positive isolates had same PFGE patterns suggested a transmission of a common strain among these workers. The detection of genes for PVL in *S. aureus *obtained from food handlers is of public health interest since these food handlers can serve as sources of transmission of PVL producing *S. aureus *in the community especially among family members.

Given that the majority of the isolates in this study contained genes for an array of virulence factors, they are potential causes of serious *S. aureus *infections. Therefore food handlers carrying these strains can contaminate food that can lead to food poisoning [7]. Thus, our results support the current practice of screening restaurant workers to identify *S. aureus *carriers and referring them to the appropriate health authorities for decolonization. Although the *S. aureus *carriage rate and their antibacterial resistance in this study may be similar to those found among healthy individuals in the general population [30], it is important to decontaminate food handlers carrying *S. aureus *because their exposure to, and possible contamination of food prepared and served in restaurants is a public health concern.

## Conclusion

This study has provided updated data on the carriage of SE and other virulence genes, and initial information on the prevalence of antibacterial resistance in *S. aureus *obtained from food handlers in restaurants in Kuwait and in the Gulf region. Our results should contribute to better management of *S. aureus *carriers among the food handlers and enhance the safety of restaurant customers.

## Competing interests

The authors declare that they have no competing interests.

## Authors' contributions

All authors conceived the study. SAM isolated and performed initial characterization of the isolates. EEU and MJA performed molecular characterization of the isolates. All authors participated in the preparation of the manuscript.
